# Airway Epithelium Senescence as a Driving Mechanism in COPD Pathogenesis

**DOI:** 10.3390/biomedicines11072072

**Published:** 2023-07-23

**Authors:** Georgia Bateman, Hong Guo-Parke, Aoife M. Rodgers, Dermot Linden, Melanie Bailey, Sinéad Weldon, Joseph C. Kidney, Clifford C. Taggart

**Affiliations:** 1 Airway Innate Immunity Research Group, Wellcome Wolfson Institute for Experimental Medicine, School of Medicine, Dentistry and Biomedical Sciences, Queens University Belfast, Belfast BT9 7AE, UK; 2 Department of Respiratory Medicine, Mater Hospital Belfast, Belfast BT14 6AB, UK

**Keywords:** COPD, airway epithelial cells, cellular senescence, pathogenesis, SASP

## Abstract

Cellular senescence is a state of permanent cell cycle arrest triggered by various intrinsic and extrinsic stressors. Cellular senescence results in impaired tissue repair and remodeling, loss of physiological integrity, organ dysfunction, and changes in the secretome. The systemic accumulation of senescence cells has been observed in many age-related diseases. Likewise, cellular senescence has been implicated as a risk factor and driving mechanism in chronic obstructive pulmonary disease (COPD) pathogenesis. Airway epithelium exhibits hallmark features of senescence in COPD including activation of the p53/p21WAF1/CIP1 and p16INK4A/RB pathways, leading to cell cycle arrest. Airway epithelial senescent cells secrete an array of inflammatory mediators, the so-called senescence-associated secretory phenotype (SASP), leading to a persistent low-grade chronic inflammation in COPD. SASP further promotes senescence in an autocrine and paracrine manner, potentially contributing to the onset and progression of COPD. In addition, cellular senescence in COPD airway epithelium is associated with telomere dysfunction, DNA damage, and oxidative stress. This review discusses the potential mechanisms of airway epithelial cell senescence in COPD, the impact of cellular senescence on the development and severity of the disease, and highlights potential targets for modulating cellular senescence in airway epithelium as a potential therapeutic approach in COPD.

## 1. Introduction

Chronic obstructive pulmonary disease (COPD) is a common chronic lung disorder. COPD is characterized by persistent low grade pulmonary inflammation, progressive airflow limitation, and irreversible parenchymal lung tissue destruction [1]. Chronic obstructive bronchitis and pulmonary emphysema are key manifestations in COPD pathogenesis, the result of a vicious cycle of chronic inflammation, protease/antiprotease imbalance, and oxidative stress [1,2,3]. COPD has become the third leading cause of mortality worldwide, claiming 3.23 million lives in 2019 [2]. Despite decades of research, there is currently no effective treatment for COPD. Long-term tobacco smoke exposure is the predominant risk factor associated with COPD. Other risk factors include pollutants and occupational exposure to noxious agents, host genetic factors, history of asthma, and childhood respiratory viral infections and social deprivation [1,2,3,4]. In addition, mounting evidence suggests that accelerated lung aging plays an important role in the onset and development of the disease [2,5,6,7,8]. The incidence of COPD is 5–6 times higher in the elderly, affecting 1 in 10 people over 45 [1,2,3,4]. Interestingly, the prevalence of asthma–COPD overlap syndrome is also increased with age [5]. Hereto, there is a link between ageing and COPD etiology [2,3,4]. Given the increased life expectancy in modern society, COPD is becoming a global pandemic with increased mortality and morbidity, representing a major healthcare burden [1,2,4].

Cellular senescence refers to a state of irreversible and permanent cell cycle arrest in which cells gradually lose their normal physiological function and proliferative capacity [9,10,11,12]. Consequently, cellular senescence results in impaired tissue repair and remodeling, loss of physiological integrity, organ dysfunction, and increased susceptibility to diseases [11,12,13,14]. Despite proliferative arrest, senescent cells remain metabolically active. They release secretomes called senescence-associated secretory phenotype (SASP), including proinflammatory cytokines, chemokines, growth factors, and matrix metalloproteinases (MMPs) [5,6,7,11,12]. These mediators further sustain senescence in an autocrine and paracrine manner, thus either amplifying senescence in themselves or/and spreading senescence to their neighboring cells [11]. Although cellular senescence plays a positive role in wound healing and cancer prevention, it is also involved in the pathogenesis of many age-related diseases including COPD [3,4,5,6,7,8,9]. The accumulation of senescent cells, and, therefore, SASP factors, in damaged tissues also results in dysregulated innate and adaptive immune responses to pathogens (immunosenescence) and chronic low-grade inflammation due to the proinflammatory cytokine response (inflammaging) [9,11].

As a physical barrier between the internal and external environment, airway epithelial cells play an important role in maintaining lung homeostasis and host defense in response to a legion of exogenous stressors and pathogens [15,16]. Airway epithelium morphologic and functional abnormalities have been implicated in the pathogenesis of many chronic inflammatory pulmonary disorders including COPD, asthma, and cystic fibrosis [17,18,19]. In line with airway epithelium senescence, chronic inflammation, oxidative stress, and protease dysregulation have also been linked to the pathological changes in COPD. These include goblet cell hyperplasia, mucus hypersecretion, emphysema, and small airway fibrosis [5,6,7,8,9,20,21,22]. Given the multiple overlapping characteristics between COPD chronic inflammation and cellular senescence, the molecular mechanisms underlying airway epithelium senescence in the initiation, development, and progression of COPD still remains incompletely understood. The focus of this review is on airway epithelial cellular senescence and the associated signaling pathways implicated in the pathogenesis of COPD. The possibility of manipulating airway cellular senescence as a novel intervention in the management of COPD will also be discussed.

## 2. Cellular Senescence and the COPD Airway Epithelium

Normal lung ageing involves both physiological and structural changes, leading to the disruption of tissue homeostasis. Progressive decline in lung function with age is caused predominantly by narrowing of the peripheral airways, decreased lung elasticity, and enlargement of the alveolar spaces, so-called senile emphysema [3,7,8]. Elevated levels of reactive oxygen species (ROS), low-grade inflammation, shortened telomeres, and increased senescence are characteristics of the normal aging lung [3,4,5,6,7,8]. These features also cause the aged lung to be more susceptible to damage, by both environmental stressors and infections [3,4,5,6,7,8,9].

Elevated expression of various senescence markers have been demonstrated in lung epithelial cells of smoke-induced inflammation in animal models and in COPD patients [22,23,24,25]. Accelerated lung aging, accumulation of senescent cells and increased SASP have been recognized to be important drivers in COPD lung inflammation and emphysema [6,7], affecting the progression, severity, and prognosis of the disease [13,20,21,22,23,24,25]. Overexpression of p21WAF1/CIP1 and p53 have also been shown to induce bronchial club cell senescence in both COPD patients and an in vivo model of COPD, leading to impaired airway regeneration and sustained airway inflammation [26,27].

## 3. Mechanisms of Airway Epithelial Cellular Senescence in COPD

**Figure 1:**
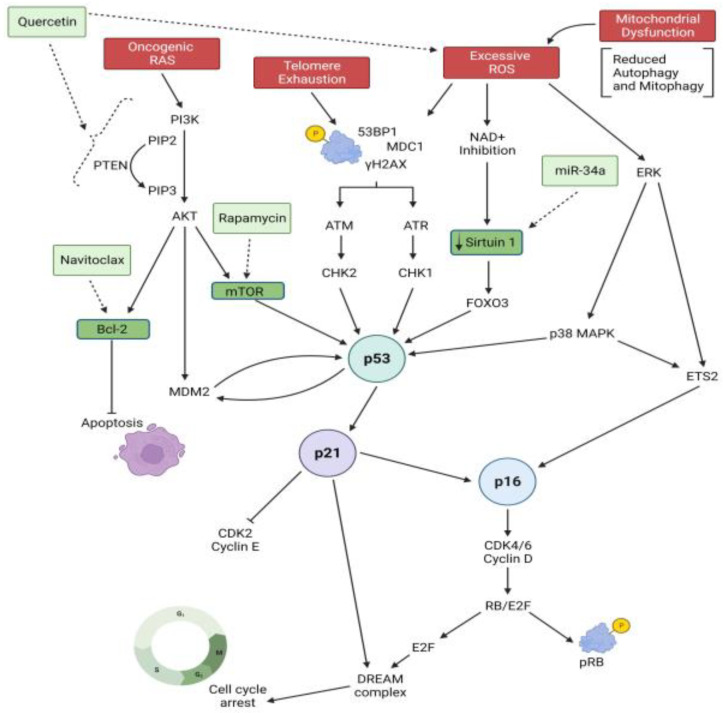
Mechanisms of airway epithelium senescence in COPD and potential therapeutic targets. Created using BioRender.com. Red boxes and solid lines represent senescence inducers and senescence signaling pathways. Light and darker green boxes and dotted lines represent potential therapeutic approches and drugs targeting senescence.

Cellular senescence is largely regulated through two key canonical senescence-inducing pathways, the p53/p21WAF1/CIP1 and p16INK4A/RB (retinoblastoma) pathways. These pathways can both interact and work independently to mediate cell cycle arrest [6,7,8,9,11,28]. Both p21WAF1/CIP1 and p16INK4A are cyclin-dependent protein kinase (CDKs) inhibitors. They trigger cell-cycle arrest by inhibiting RB phosphorylation, leading to accumulation of the active form of RB [6,7,8,9]. Both pathways involve the complex interaction and crosstalk of multiple upstream and downstream mediators. It has been suggested that p21WAF1/CIP1 is essential in the initiation of senescence, whereas p16INK4A plays an important role in sustaining cellular senescence at a late stage [11]. Figure 1 provides a summary of the heterogeneous intrinsic and extrinsic signals and pathways involved in mediating airway epithelial cellular senescence in COPD. DNA damage triggered by telomere shortening or oxidative stress arrests the cell cycle via a persistent DNA damage response (DDR). A continuous DDR is mainly initiated by ataxia-telangiectasia mutated (ATM) and Rad3-related protein (ATR) kinases, leading to the formation of DNA-damage protein foci such as phosphorylated histone H2AX (γH2AX), 53BP1, and MDC1 (mediator of DNA damage checkpoint 1) [5,6,7,8,9]. The phosphorylation of these kinases and their downstream kinases, checkpoint kinase 2 (CHK2) and 1 (CHK1), activate the DDR through the canonical p53/p21WAF1/CIP1 pathway, which promotes senescence by upregulating p53 and its downstream transcriptional regulator p21WAF1/CIP1 [11,28]. Consequently, p21WAF1/CIP1 inhibits cyclin E–Cdk2 and RB inactivation, leading to cell cycle arrest. In addition, p21WAF1/CIP1 also inhibits cyclin–cyclin-dependent kinase complexes that block the formation of the DREAM complex, which represses cell-cycle genes by binding to their homology region [11]. Unlike DDR-induced senescence, oxidative stress and epigenetic modifications promote senescence by activating p16INK4a/pRB signaling [4,9,11]. Upregulated p16INK4a prevents the formation of cyclin D–CDK4/6 complexes and the phosphorylation of RB, thus promoting the formation of the RB–E2F complex and cell-cycle arrest. In parallel, this pathway can also act via the ERK/ETS1/2 or p38MARK pathways which upregulates ETS2 and in turn activates p16INK4a. Moreover, p16INK4a can be upregulated by inhibiting NAD+, leading to the reduced expression of sirtuin 1 (SIRT1) and activation of FOXO3, p53, and RelA/p65 [11,28]. Oxidative stress also activates the mTOR (mammalian target of rapamycin) pathway by inhibiting PTEN (phosphatase and tensin homolog) to activate the oncogenic RAS-PI3K (phosphoinositide-3-kinase)/AKT signaling or by downregulating the activation of AMPK (AMP kinase) [11,28].

### 3.1. DNA Damage-Induced Senescence on COPD Airway Epithelium

Described by Hayflick and Moorhead in 1961, cellular senescence was first observed when cultured human fibroblasts showed a finite proliferative capacity known as “telomere-dependent replicative senescence” [9,10,11]. Telomeres are DNA structures stabilized by a complex of shelterin proteins which prevent chromosome ends from degradation [6,7,8,9,10,29,30]. Telomeres become progressively shorter with repeated cell division until they eventually reach a critical threshold termed the Hayflick limit, leading to the activation of DDR [29,30]. When DNA damage is beyond repair, cellular replication becomes exhausted, resulting in permanent cell cycle arrest and subsequent cellular senescence [6,7,8,9,10,30].

In COPD, chronic CS-exposure-induced oxidative stress may contribute to telomeric DNA damage, promoting cellular senescence, emphysema, and inflammation in the lung [29,30]. Lung cells from COPD patients demonstrate increased global and telomeric DNA damage [30,31,32]. Shortened leukocyte telomere length has been linked to smoke-induced accelerated lung ageing, lung function deterioration, and increased risk for the development of COPD [32,33]. Walters et al. suggested that CS accelerates aging of the small airway epithelium by dysregulation of age-related gene expression and enhanced telomere erosion [34]. Ahmad et al. showed that the disruption of the shelterin complex protein TPP1 (telomere protection protein 1) contributed to persistent telomeric DNA-damage-induced airway epithelial cell senescence in mice with emphysema as well as in smokers and COPD patients [35]. Voic et al. identified 243 common gene expression changes during replicative senescence and CS exposure in human airway epithelial cells [36]. They found that molecular pathways altered by CSs including ROS, proteasome degradation, and NF-κB signalling may promote replicative senescence in airway epithelium [36].

CS is the most well-studied extrinsic inducer of oxidative DNA damage [30,37]. Oxidative DNA damage and repair pathways play an important role in the onset and development of COPD [36,37,38]. Tzortzaki et al. proposed the airway epithelium DNA damage hypothesis in the initiation of COPD [38]. They suggested that CS induces persistent oxidative stress leading to oxidative DNA damage of the lung epithelial barrier cells, which contributes to the abnormal inflammatory and host immune response in COPD [38].

DNA double-strand breaks (DSBs) are among the most detrimental types of DNA damage caused by CS [30]. Oxidative-stress-induced impaired repair of DNA DSBs contribute to COPD pathogenesis via the induction of apoptosis, cell senescence, and pro-inflammatory responses [30,37,38]. Phosphorylated histone H2AX (γH2AX), a sensor of DSB, can recruit DNA repair protein foci including phosphorylated ATM/ATR substrates and phosphorylated 53BP1 to the site of DNA damage [11,37,38,39]. Accelerated alveolar type-2 epithelial cell senescence coupled with increased γH2AX, DSB foci, phosphorylated NF-kB, and IL-6 has been demonstrated in smokers with COPD compared to smokers without COPD [31]. These results indicate oxidative DNA damage contributes to COPD pathogenesis, at least in part, by inducing cell senescence and proinflammatory responses [31].

The histone deacetylases (HDACs) 1 and 2, regulate oxidative-stress-induced DNA damage and repair machinery and cellular senescence via an epigenetic mechanism [39,40]. HDAC2, a critical marker of chromatin remodeling, is reduced as a consequence of oxidative-stress-mediated DNA damage and impaired repair in lung parenchyma, bronchial biopsies in COPD patients [38]. HDAC2 and SIRT1/6 (NAD+-dependent protein/histone deacetylases) are important regulators of inflammation, histone/DNA epigenetic modifications, DDR, and cellular senescence [40,41,42,43,44]. Decreases in HDAC2 and SIRT1/6 expression are demonstrated in diseases including COPD [41,42,43,44]. CS causes persistent DNA damage and cellular senescence via the HDAC2-dependent mechanism leading to COPD/emphysema [34,40].

Oxidative DNA damage, DSBs, and HDAC2 posttranslational modifications have recently been shown to induce DDR [40,45,46,47]. Consequently, this results in chronic inflammation in COPD due to the aberrant SASP response. HDAC2 activity has been linked to increased acetylation of nuclear respiratory factor 2 (Nrf 2), a transcription factor that promotes the production of cellular antioxidants and detoxifying enzymes [46]. Another DNA double-strand break repair marker Ku86 was found to be decreased in the parenchymal lung tissue of COPD patients, including small airways [8,48]. Downregulation of lamin B1 nuclear protein, a regulator of genomic stability, has also been recognized as a hallmark for the development of full senescence [7]. The reduced lamin B1 has been demonstrated to contribute to the progression of senescence in COPD pathogenesis through the aberrant mTOR pathway [5,6,7,8,9].

### 3.2. Oxidative-Stress-Induced Senescence on COPD Airway Epithelium

Oxidative stress due to elevated levels of ROS and/or impaired antioxidant defense contributes to COPD pathogenesis by inducing protease/antiprotease imbalance, mucus hypersecretion, membrane lipid peroxidation, and mitochondrial dysfunction. It also results in alveolar epithelial injury, remodeling of the extracellular matrix, altered apoptosis, and cellular DNA damage [45,49]. Elevated NF-κB expression and activation of airway epithelial cells and macrophages have been shown in COPD patients due to oxidative-stress-induced SASP secretion [44,47,49]. However, the exact mechanism of oxidative-stress-induced airway epithelium senescence is still largely unknown.

Cigarette smoke contains thousands of toxic chemical substances including excessive oxidants and reactive oxygen species (ROS) which injure lung epithelial cells and connective tissues in smokers [4,37,45,49,50,51]. Epithelial cell injury products induce epithelial-derived inflammatory mediators which in turn secrete proteolytic enzymes and more ROS, leading to further damage to lung tissue [49,50,51]. The activation of the antiageing SIRT1 1/FOXO3 pathway seems to protect against emphysema through FOXO3-mediated reduction of cellular senescence in emphysematous mice [42]. Baker et al. demonstrated that accumulation of H_2_O_2_ selectively upregulates microRNA-34a in bronchial epithelial cells of COPD patients via the PI3K/AKT/mTOR pathway by inhibiting SIRT1 and SIRT6. This further promotes oxidative stress, inflammation, and cellular senescence [43]. Impaired Nrf2 activity and Nrf2-mediated antioxidant gene expression have been found in aged bronchial epithelial cells in COPD patients, that increase the expression of genes involved in inflammation, secretion of airway mucus, and inactivation of antiproteases [52,53,54].

Mitochondria is the primary source of ROS. ROS-induced mitochondrial structural and functional abnormality is a hallmark characteristic of CS-induced senescence in the lung [44,55]. Damaged mitochondria, in turn, further promote mitochondrial ROS production [49,50]. Mitochondrial biogenesis is controlled by transcriptional coactivator PGC-1a, peroxisome proliferator-activated receptor γ coactivator-1β (PGC-1β), the activation of Nrf2, AMPK signaling, and SIRT1 regulation, resulting in enhanced ROS and cellular senescence [20,23,51]. Mitochondrial dysfunction due to oxidative stress/redox imbalance and aberrant mTOR activation has been implicated in COPD epithelial cells, at least in part contributing to accelerated cellular senescence in the lung [51].

### 3.3. Reduced Autophagy

Autophagy is a process of proteostasis in which the cells respond to stress-induced damage through lysosome-dependent degradation of damaged proteins and organelles [56,57,58,59]. Autophagy responds to energy decline through AMP-activated protein kinase (AMPK) and is negatively regulated by growth factor stimulation through the PI3KC1/AKT/mTORC1 pathway [58,59]. Mitophagy is a selective type of autophagy for the specific lysosomal degradation of damaged mitochondria [60,61]. Both autophagy and mitophagy reduce with age, resulting in a reduced capacity to alleviate the consequences of oxidative stress and increased cellular senescence [58,59,60,61]. Impaired autophagy leads to the accumulation of oxidative-stress-induced damage proteins or organelles and has been implicated in accelerating lung ageing and COPD-emphysema exacerbations and pathogenesis [62,63,64,65,66]. Fujii and colleagues reported that autophagy in response to CS exposure was significantly decreased in human bronchial epithelial cells from COPD patients with an increased accumulation of p62, a marker of autophagy inhibition [54]. Bodas et al. demonstrated that CS induces bronchial epithelial cell apoptosis and senescence via ROS-mediated autophagy impairment [67]. Insufficient autophagic clearance of damaged proteins, including ubiquitinated proteins and lamin B1, is involved in promoting bronchial epithelial cell senescence and COPD-emphysema pathogenesis [56,64,65,66].

In COPD, impaired autophagy leads to impaired mitophagy, in which damaged mitochondria persistently accumulate in the cells, contributing to increased susceptibility to cellular senescence [62,63,64,68]. CS induces mitophagy through the stabilization of the mitophagy regulator PINK1 and recruitment of PARK2 [51]. In COPD bronchial epithelium, a lower level of PARK2 and increased PINK1 may reflect an accumulation of damaged mitochondria due to insufficient mitophagy, leading to a decline in mitochondrial membrane potential and increased mitochondrial ROS production [67,69]. CS contributes to cellular senescence in COPD likely via ROS-induced mitochondrial dysfunction, mitochondrial DNA damage, and impaired mitophagy mediated by the PINK1-PARK2 pathway [65,67,68,69,70].

### 3.4. SASP Further Accelerates Senescence and COPD Inflammation

The proinflammatory SASP exhibited by senescent cells is a hallmark of senescence which may contribute to the chronic low-grade inflammation in COPD pathogenesis [3,5,9]. SASP comprises proinflammatory cytokines, growth factors, MMPs, proteases, and extracellular vesicles (EVs) that have the potential to alter their surrounding tissue microenvironment and reinforce senescence in a paracrine and autocrine manner [5,6,12,71]. COPD epithelial cell senescence has been linked to increased activation of NF-κB signaling, the predominant transcription regulator of inflammation. It also promotes a robust and persistent production of certain cytokines, including IL-6, IL-8, IL-1α/β, TNFα, and CXCL1 [9,12,72]. Epithelial-derived SASP may partially explain the persistent chronic inflammation observed even after smoking cessation and the increased baseline secretion of inflammatory mediators in the COPD lung [3,5]. Kumar et al. termed inflammatory mediators observed in COPD inflammation as the “COPD-associated secretory phenotype” (CASP) [71]. They found a striking overlapped profile between CASP and SASP in terms of proinflammatory cytokines, chemokines, growth factors, and MMPs, indicating a causative role of SASP in COPD pathogenesis [71].

**Figure 2:**
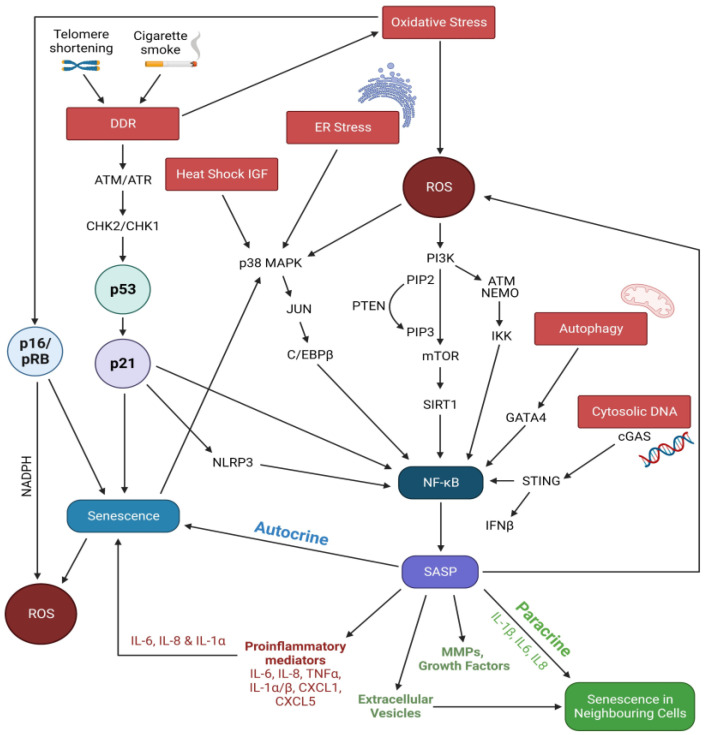
Possible mechanisms of SASP regulation on COPD airway epithelium. Created using BioRender.com. Red and dark red boxes represent key inducers that mediate the secretions of SASP through differenct signal pathways in an autocrine manner. Green writings and box represent mediators that are involed in spreading senesence to neighbouring cells in a paracrine manner.

SASP is mainly regulated through the activation of NF-κB and C/EBPB (CCAAT/enhancer-binding protein-β) via both DDR-dependent p21WAF1/CIP1 and DDR-independent p16INK4a pathways [73,74,75,76,77] (Figure 2). Persistent DDR activates p21WAF1/CIP1 and NF-κB through the stress-inducible kinase p38/MAPK, JAK (Janus-activated kinase) has been demonstrated as the major mechanism of SASP production [73,74,75,78]. Oxidative-stress-induced DDBs lead to the production of IL-1a, IL-6, IL-8, TNFα, GM-CSF, GROα, VEGF, TGFβ, MCP-1, and MMP-1, -8, and -9 [3,47,72,79]. ROS can activate NF-κB-mediated SASP response not only by promoting nuclear translocation of NEMO (NF-κB essential modulator) through the activation of ATM but also by inhibiting SIRT 1 via activation of the PI3K/AKT/mTOR pathway and increased MMP-9 [4,44,80,81]. More recently, CS-associated DNA damage has been found to cause self-DNA release leading to the accumulation of cytoplasmic DNA in the cytosol [82,83]. Of note, cytoplasmic DNA accumulation has been found to trigger aberrant activation of the cGAS-STING cytoplasmic DNA sensing pathway and promote the SASP either via activation of NF-κB or via induction of interferon-β [82,83]. Excess oxidative stress and DNA damage also triggers the activation of the NLRP3 inflammasome, stimulating NF-κB and the IL-1β-mediated inflammatory cascade, thus further perpetuating the prolonged low-grade inflammatory response in COPD epithelium [4,84].

Activation of the DDR-independent p16INK4a pathway also activates NADPH oxidases, resulting in increased oxidative stress, which further activates NF-κB [51]. Loss of proteostasis mediated by heat shock proteins (HSP) and endoplasmic reticulum (ER) stress can also activate the NF-κB–C/EBPβ pathway via p38MAPK signaling [4,5,85]. Impairment of autophagy hampers degradation of the transcription factor GATA4, which activates NF-κB and leads to initiation of the inflammatory cascade involving the NF-κB, IL-1α, TGF-β, and IL-6 [73].

## 4. Therapeutics Targeting Senescence

There is currently no known cure for COPD; however, there are therapies available to mitigate the complications associated with COPD, alleviate symptoms, and generally increase quality of life. These include smoking cessation, bronchodilators, corticosteroids, pulmonary rehabilitation, and, in severe cases, lung transplantation [86]. While these treatments alone or in combination are often able to alleviate symptoms, they do not target the underlying disease process, including a dysregulated repair response and inflammation. More recently, therapies specifically targeting senescent cells have been studied, in particular, senolytics that work to delete senescent cells and senostatics that act to inhibit the pathways leading to cellular senescence (Figure 1, dotted lines).

### 4.1. Inhibition of Prosurvival Pathways

Senolytics are compounds capable of inducing apoptosis in senescent cells, whilst having minimal to no effect on surrounding proliferating cells. Many of the senescence triggers also induce apoptosis; senescent cells have developed an ability to avoid apoptosis via the induction of prosurvival/antiapoptosis pathways. Despite these cells being primed for death, they have an enhanced resistance to apoptosis through these pathways. However, senescent cells also have an increased sensitivity to the inhibition of antiapoptotic pathways and are heavily dependent on these pathways to ensure their survival. This increased sensitivity and dependency highlights a promising target for the removal of senescent cells through inhibition of antiapoptotic pathways.

The BCL protein family plays a vital role in the regulation of cell death, through both apoptosis and autophagy [87]. Targeting this family has been extensively studied in pharmacological interventions for multiple cancers [88]. The small-molecule inhibitor ABT- 263, known as Navitoclax, is a pan Bcl-2 inhibitor, targeting Bcl-2, Bcl-xl, and Bcl-w. This inhibitor has previously been shown to selectively kill senescent cells through the induction of apoptosis in various cell types [89,90]. However, despite these promising results, Navitoclax can cause severe side effects. In a previous phase I dose-escalation study in patients with lymphoid malignancies, hematological toxicities such as thrombocytopenia and, less commonly, neutropenia were reported as platelet survival is dependent on the BCL pathway [91]. Overall, this evidence suggests that, despite successful removal of senescent cells through BCL inhibition, the side effects will likely limit its therapeutic availability.

Quercetin has been shown to reduce oxidative stress and inflammation in an elastase/LPS COPD-like mouse model [92]. This plant polyphenol has more recently been linked to senescence in the lung, interacting with the PI3K/AKT prosurvival pathway, and has been shown to mitigate fibrosis, reduce the presence of senescence markers and SASP, and induce apoptosis of senescent fibroblasts in an in vivo IPF model [93].

The first human senolytic clinical trial involved quercetin, given in combination with dasatinib (D + Q) to patients with idiopathic pulmonary fibrosis (IPF). In this trial, 14 stable IPF patients were recruited and given D + Q; they showed statistically significant and clinically meaningful increases in physical function, and D + Q therapy was overall well tolerated. This trial provided initial evidence that D + Q therapy could alleviate the negative symptoms associated with cellular senescence; however, this would have to be studied further as circulating SASP factor analysis was inconclusive in this study. D + Q would also have to be studied in a much larger cohort before clinical use.

### 4.2. Inhibition of mTOR

As discussed previously, the serine/threonine kinase mTOR is an important downstream effector of the PI3K/AKT pathway and plays important roles in proliferation, survival, and autophagy, as well as induction and maintenance of the SASP. The PI3K/AKT pathway has also previously been linked to the corticosteroid resistance seen in many COPD patients, a factor that prevents inhaled corticosteroids from effectively reducing disease progression and mortality [94]. This led to the hypothesis that inhibitors of mTOR would successfully suppress the SASP response and potentially increase susceptibility to inhaled corticosteroids. A study by Harrison et al. in 2009 showed that the mTOR inhibitor rapamycin was able to extend the lifespan of both aged female and male mice, even when administered at later life [95]. Rapamycin has also been shown to restore corticosteroid sensitivity in COPD. However, rapamycin has adverse effects including anemia, mouth ulcerations, and delayed wound healing, alongside significant suppression of the immune system. Whilst the lifespan of mice was prolonged in a pathogen-free environment, this level of immune suppression could become problematic elsewhere [96]. These substantial side-effects prevent rapamycin from being a long-term solution or a preventative measure in healthy individuals.

Metformin (MET) is an oral hypoglycemic drug widely used worldwide in type-2 diabetes mellitus treatment. More recently, MET has been suggested as a promising therapeutic for various inflammatory diseases [97]. MET has anti-inflammatory properties, including a reduction in mitochondrial ROS production and IL-6 release from alveolar macrophages. MET induces autophagy through AMPK activation, and subsequent mTOR inhibition [98]. As this is already a widely used drug, the repurposing of MET as an inhibitor of mTOR provides a promising avenue for COPD therapy.

### 4.3. Micro-RNAs

Micro-RNAs (miRNA) are small noncoding RNAs that regulate the gene expression of target genes. The study of miRNAs is extensive due to their involvement in a wide range of biological processes, including apoptosis and ageing. Increasing evidence shows miRNAs play a role in the regulation of multiple proteins involved in senescence, including p16, p53, and SIRT1.

Micro-RNA-34a (miR-34a) is a p53-regulated tumor suppressor miRNA that has been shown to be involved in controlling apoptosis, cell cycle arrest, and senescence. It has previously been shown that miR-34a is an important downregulator of the antiageing molecule SIRT1 and, to a lesser extent, SIRT6 [99]. Levels of miR-34a are significantly elevated in lung homogenates, epithelial cells, and sputum from severe COPD cases, indicating the role of miR-34a in the senescence seen in COPD. Inhibiting miR-34a with a specific antagomir restores SIRT1 and SIRT6 levels in COPD epithelial cells, alongside a significant decrease in markers of cellular senescence, including inflammatory SASP factors [100].

### 4.4. Extracellular Vesicles

Extracellular vesicles (EVs) are nanosized lipid bilayer vesicles that are universally released by all studied cell types, allowing for their detection in all tested bodily fluids, including sputum, serum, urine, and bronchoalveolar lavage fluid. EVs are mediators of cell–cell communication and are able to transfer their content to recipient cells either via direct contact or through internalization by recipient cells.

EVs are able to spread senescence through their content, including miRNAs and proteins, both to surrounding cells within the lung and through the circulatory system to other organs, potentially explaining the comorbidities seen in COPD [100]. Despite EVs having the ability to propagate senescence and inflammation, the use of EVs as biomarkers and potential therapeutic intervention options is something that is currently being extensively studied [100,101,102,103].

The use of EVs derived from healthy, nonsenescent cells, mainly from mesenchymal stem cells (MSCs), has shown promising results in decreasing senescence levels, both in vitro and in vivo [101,102,103]. EVs have also been studied in the context of drug-delivery systems. Enclosing drugs in EVs allows for specific cell-targeted delivery with increased stability [104]. This is currently under investigation predominantly in cancer therapies, and faces many barriers before entering common practice, including EV isolation standardization, protocols for successful administration, and technique for loading EVs with desired content [105].

### 4.5. Telomere Shortening

As previously discussed, telomere shortening is a key factor in the aging of an organism, as it naturally occurs as a consequence of cell division [106]. Therapies to combat telomere shortening are currently being studied. These include activators of telomerase, the enzyme responsible for maintaining telomere length, activators of telomerase reverse transcription (TERT), and intracellular administration of TERT mRNA [107]. However, it should be noted that telomere therapies should be taken with caution, as off-target effects and hyperactive telomerase can promote cancer development.

Whilst the complex pathways involved in cellular senescence provide many available targets for therapeutic options, this also presents with its own issues. Due to extensive crosstalk between the senescence pathways, the modulation or inhibition of one branch could just promote the activation of another. Senescence is a key process in many homeostatic functions, particularly tumor suppression, as previously stated; therefore, the use of therapeutics to target senescence has to be closely monitored to ensure there are no detrimental or off-target effects. Finally, there has to be an appreciation that aging and senescence can affect cells differently, and the variability between cell types means senescence is studied predominantly in a cell-type specific manner. This is important to note as a therapeutic that presents with beneficial findings in one cell-type may not be viable due to its detrimental effect in another.

## 5. Conclusions

COPD is defined by progressive and irreversible airflow limitation, associated with an enhanced inflammatory response within the lungs. There is increasing evidence to suggest that cellular senescence is a key mechanism involved in COPD pathogenesis. Cellular senescence present in the COPD epithelium has been shown to be associated with many of the features of COPD pathogenesis, including chronic inflammation, mitochondrial dysfunction, and dysregulated repair mechanisms. The release of the SASP mediators from senescent cells is also able to further promote senescence, both in neighboring cells and throughout the body through the circulatory system, potentially explaining the co-morbidities associated with COPD. These comorbidities also often overlap with age-related diseases, sharing mechanisms and further highlighting COPD as a disease of accelerated age. So called ‘antiaging’ therapies to target senescence pathways, or the removal of senescent cells via apoptosis, are in development; however, the complex pathways involved in senescence and the downstream effects make it difficult to target. EVs have promising therapeutic potential in targeting senescence, due to the ability to manipulate their content. Clinical trials of these therapies are significantly challenging, as COPD is progressive with multiple mechanisms of disease; thus, treatments have to be monitored over long periods of time in large cohorts for valid results. Further understanding of the mechanisms involved in senescence, and their overlap and contribution to the development of COPD pathogenesis, will aid in the development of novel therapies that could reverse the disease process and prevent disease progression.

## Data Availability

Not applicable.
